# The Impact of Low-Level Lead Toxicity on School Performance among Hispanic Subgroups in the Chicago Public Schools

**DOI:** 10.3390/ijerph13080774

**Published:** 2016-08-01

**Authors:** Michael J. Blackowicz, Daniel O. Hryhorczuk, Kristin M. Rankin, Dan A. Lewis, Danish Haider, Bruce P. Lanphear, Anne Evens

**Affiliations:** 1 Division of Epidemiology and Biostatistics, University of Illinois at Chicago School of Public Health, Chicago, IL 60612, USA; krankin@uic.edu; 2 Center for Global Health, University of Illinois College of Medicine, Chicago, IL 60612, USA; dhryhorc@uic.edu (D.O.H.); danish.haider@gmail.com (D.H.); 3 Division of Environmental and Occupational Health Sciences, University of Illinois at Chicago School of Public Health, Chicago, IL 60612, USA; 4 School of Education and Social Policy, Northwestern University, Evanston, IL 60208, USA; dlewis@northwestern.edu; 5 Child & Family Research Institute, BC Children’s Hospital and Faculty of Health Sciences, Simon Fraser University, Vancouver, BC V5Z 4H4, Canada; blanphear@sfu.ca

**Keywords:** lead poisoning, school performance, Hispanics

## Abstract

*Background*: Environmental lead exposure detrimentally affects children’s educational performance, even at very low blood lead levels (BLLs). Among children in Chicago Public Schools (CPS), the severity of the effects of BLL on reading and math vary by racial subgroup (White vs. Hispanic vs. non-Hispanic Black). We investigated the impact of BLL on standardized test performance by Hispanic subgroup (Mexican, Puerto Rican, and Other Hispanic). *Methods*: We examined 12,319 Hispanic children born in Chicago between 1994 and 1998 who were tested for BLL between birth and 2006 and enrolled in the 3rd grade at a CPS school between 2003 and 2006. We linked the Chicago birth registry, the Chicago Blood Lead Registry, and 3rd grade Illinois Standard Achievement Test (ISAT) scores to examine associations between BLL and school performance. Primary analyses were restricted to children with BLL below 10 µg/dL (0.483 µmol/L). *Results*: BLLs below 10 µg/dL (0.483 µmol/L) were inversely associated with reading and math scores in all Hispanic subgroups. Adjusted Relative Risks (RR_adj_) and 95% confidence intervals (CI) for reading and math failure were 1.34 (95% CI = 1.25, 1.63) and 1.53 (95% CI = 1.32, 1.78), respectively, per each additional 5 µg/dL of lead exposure for Hispanic children; RR_adj_ did not differ across subgroups. We estimate that 7.0% (95% CI = 1.8, 11.9) of reading and 13.6% (95% CI = 7.7, 19.2) of math failure among Hispanic children can be attributed to exposure to BLLs of 5–9 µg/dL (0.242 to 0.435 µmol/L) vs. 0–4 µg/dL (0–0.193 µmol/L). The RR_adj_ of math failure for each 5 µg/dL (0.242 µmol/L) increase in BLL was notably (*p* = 0.074) stronger among black Puerto Rican children (RR_adj_ = 5.14; 95% CI = 1.65–15.94) compared to white Puerto Rican children (RR_adj_ = 1.50; 95% CI = 1.12–2.02). *Conclusions*: Early childhood lead exposure is associated with poorer achievement on standardized reading and math tests in the 3rd grade for Mexican, Puerto Rican, and Other Hispanic children enrolled in Chicago Public Schools. While we did not see interactions between BLL and ISAT performance by Hispanic subgroup, the stronger association between BLL and math failure for Black Puerto Rican children is intriguing and warrants further study.

## 1. Introduction

Hispanic children in the United States have historically been at greater risk of elevated blood lead levels (BLLs) than non-Hispanic whites, though at lower risk than non-Hispanic blacks [1,2,3]. Risk factors for elevated BLLs among Hispanics have included substandard housing [2], cultural practices (folk remedies, food preparation), parental exposures (occupation, maternal pica), and diet (contaminated candy, iron deficiency) [4,5,6]. Over the past several decades, the elimination of lead from gasoline and residential paint has dramatically reduced lead levels in all children in the United States, including Hispanics [2]. In the 2007–2010 cycles of the National Health and Nutrition Examination Survey (NHANES), the geometric mean BLLs of Mexican and non-Hispanic white children ages 1–5 years converged at 1.3 µg/dL (0.063 µmol/L), while non-Hispanic black children were significantly higher at 1.8 µg/dL (0.087 µmol/L) [3]. Despite these improvements, even very low levels of lead can cause intellectual and behavioral deficits in children, including a worsening in school performance [7,8,9,10,11,12,13].

We previously examined the effect of BLLs on the performance of 58,560 children enrolled in the 3rd grade at Chicago Public Schools (CPS) between 2003 and 2006 on the Illinois Standard Achievement Test (ISAT) [13]. We found that BLLs were inversely associated with reading and math ISAT scores and positively associated with failure rates at BLLs below 10 µg/dL (0.483 µmol/L), even after adjusting for confounders such as poverty, race/ethnicity, gender, maternal education and very low birth weight or preterm birth. The relative risks varied modestly by race/ethnicity: in adjusted analyses, the relative risk of failing the reading and math tests per 1 µg/dL (0.048 µmol/L) increase in BLL was higher among non-Hispanic whites (1.14 for reading; 1.11 for math), than among Hispanics (1.08; 1.09) and non-Hispanic blacks (1.05; 1.05). 

Although Hispanics are generally considered to be an ethnic group, they represent a heterogeneous mix of Native American, European, and African ancestries. They present a unique opportunity to disentangle the clinical, social, environmental, and genetic underpinnings of population differences in health outcomes [14]. In this paper, we extend our earlier analyses on the effects of low-level lead exposure on school performance to estimate these risks for Hispanic subgroups in our CPS cohort. 

## 2. Methods

**Figure 1:**
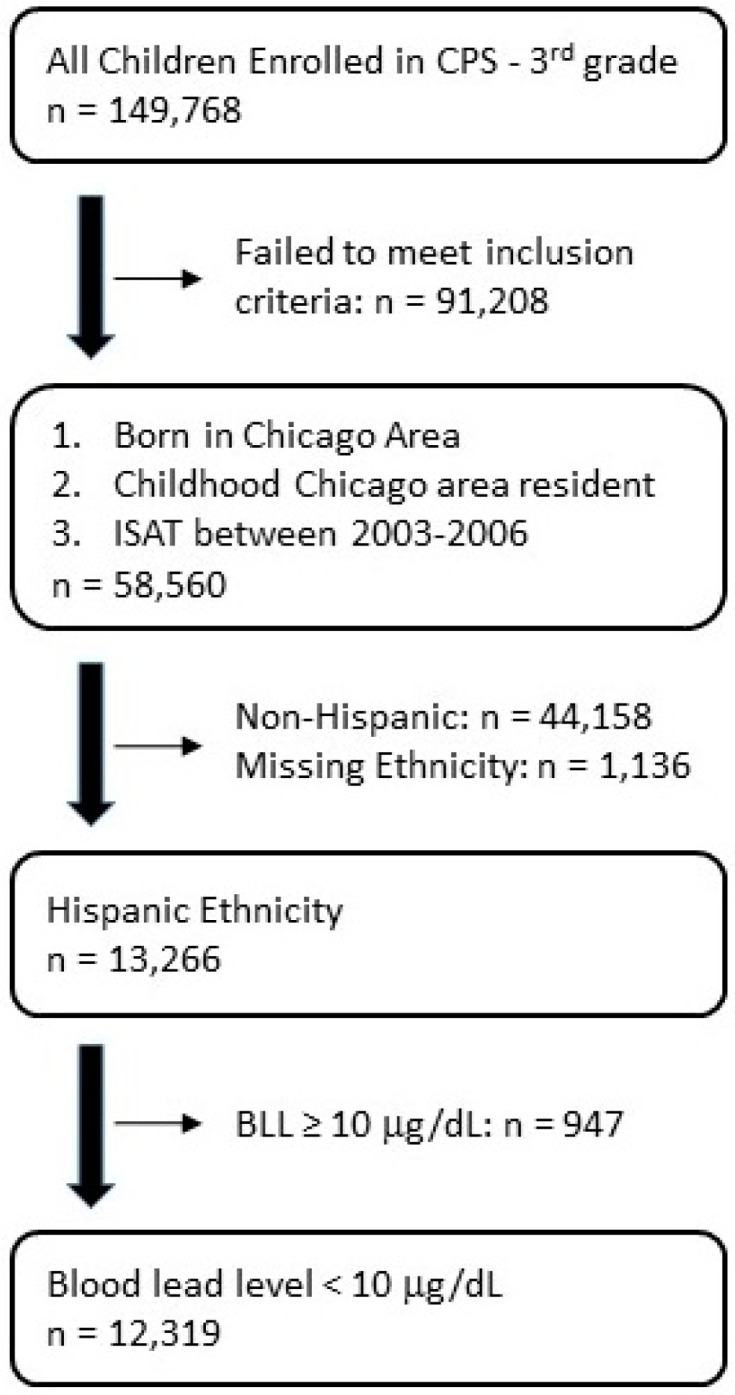
Population Flow Chart. Each subsequent sample (black border) is a subsample of the parent sample following stepwise exclusions (unbordered, below parent sample). CPS: Chicago Public Schools; ISAT: Illinois Standard Achievement Test.

We previously examined 58,650 children born in Chicago between 1994 and 1998, who were tested for blood lead between birth and 2006 and were enrolled in the 3rd grade at a CPS school between 2003 and 2006. We linked the Chicago birth registry, the Chicago Blood Lead Registry, and 3rd grade ISAT scores to examine associations between BLLs and school performance. Linkage of these three datasets was performed by Chapin Hall, Center for Children at the University of Chicago, which maintains a data sharing and data analysis agreement with the Chicago Department of Public Health (CDPH). Chapin Hall received BLL and birth registry datasets from CDPH. Chapin Hall matched these files to CPS data by first name, last name, and date of birth using probabilistic matching methods. The study population is a cohort of children who were: born in the six-county area of metropolitan Chicago between 1994 and 1998; residents of Chicago during early childhood; had a BLL test reported to the CDPH between 1996 and 2006; and were enrolled in a CPS school with ISAT scores between 2003 and 2006. Blood lead testing was always completed prior to the ISAT testing date. There were 58,560 children who met these inclusion criteria, representing 39% of the 149,768 children enrolled in CPS in the third grade. We found no significant differences in the characteristics of children in the study compared with those enrolled in Chicago Public Schools, *p* < 0.0001 level, except that Hispanic children were slightly under-represented in the study sample. This is likely because Hispanic children are much more likely to have immigrated to the US and do not have available birth registry data, thus making them ineligible for the study. We then excluded 44,158 non-Hispanic children and 1136 children who were missing race/ethnicity for a total of 13,266. The primary focus of this study was to investigate the effects of low-level lead exposure on school performance, so we restricted all analyses to the 12,319 Hispanic children who had BLL less than 10 μg/dL (0.483 µmol/L). The population flow chart is shown in Figure 1. Additional details on the design, databases, and linkage protocol can be found in our previous paper [13]. 

The main outcome variable for this study was 3rd grade ISAT scores, a measure of individual student achievement relative to the Illinois Learning Standard. The ISAT measures the achievement of students for reading and mathematics in grades three through eight, and for science in grades four and seven. The ISAT was administered by the Illinois State Board of Education, which also assesses validity of the test, and was required for all public school students in the state, serving as the principal measure of school performance. ISAT results used were for the years 2003 through 2006. ISAT scores for both reading and math range from 120 to 200. Each year, the reading and math test scores are divided into four categories: academic warning (failure), below standards, meets standards, and exceeds standards. Children who get a score in the academic warning (failure) category will be retained and required to repeat the third grade unless they are retested and score higher. We examined math and reading failure as additional outcome variables.

The following analysis was restricted to Hispanic children with BLLs less than 10 µg/dL (0.483 µmol/L) (*n* = 12,319, 92.9% of all children), because for decades this level was considered the “blood lead level of concern” by the Centers for Disease Control and Prevention, and as a result it remains a widely held belief that lower blood lead levels are not harmful and therefore there is no need to control lead exposure at lower levels. Blood lead tests were used as a measure of childhood lead exposure. Over 90% of children had only a single BLL measurement. For children with more than one BLL, the most recent venous blood lead measurement was used in the analysis. Children were considered Hispanic if the mother reported Hispanic ethnicity on the birth certificate; 8449 (69%) of these children were born to Mexican, 2564 (21%) to Puerto Rican, and 1256 (10%) to Other Hispanic mothers. Although the full distribution of BLL is log-normally distributed, the distribution of blood lead at lower levels (less than 10 µg/dL (0.483 µmol/L)) is approximately normally distributed, as are the distributions of both reading and math scores. Standard bivariate analysis methods were used to investigate crude relationships between covariates, BLLs, and test performance scores. Specifically, we generated tables to compare mean BLL, math score, and reading score, as well as the proportion of children failing the math and reading sections between covariate groups (Table 1). Statistical tests were performed to test for differences in means (*t*-test) and differences in proportions (chi-square test). Linear regression models were used to test for differences in means for nominal categorical variables and for evidence of a linear trend in ordinal variables, while log probability regression models were used similarly to test for differences and linear trends in proportions. Covariates include gender, mother’s educational attainment, low-income (subsidized school lunches vs. unsubsidized school lunches), small for gestational age (SGA), preterm birth (early PTB = 20–33 weeks, late PTB = 34–36 weeks, term birth = 37–44 weeks), child’s age at time of BLL, exam type (ISAT vs. Iowa), and Hispanic subgroup (Mexican, Puerto Rican, and Other Hispanic). The SGA variable is a binary variable representing birth weight below the 10th percentile with respect to gestational age, according to the US standard [15].

Ordinary least squares regression was used to assess crude relationships between blood lead level and both math and reading scores. Multivariable models were adjusted for gender, mother’s educational attainment, low-income, SGA, preterm birth, child’s age at time of venipuncture, exam type, and Hispanic subgroup. Effect modification by Hispanic subgroup was assessed by including an interaction product of BLL and Hispanic subgroup in each model, and a type-3 *F*-test was used to test for overall effect of the interaction term. We addressed non-linearity in the relationship between BLL and test scores by comparing the root mean square error (RMSE) in the adjusted linear BLL model (only linear BLL) with the adjusted non-linear BLL model (linear BLL with quadratic BLL term).

Log binomial regression was used to assess crude relationships between BLL and failure rate for both math and reading sections of the exam. Multivariable models were adjusted for the same covariates used in the linear regression models. Effect modification for Hispanics overall and by Hispanic subgroup was assessed by including an interaction product of BLL and Hispanic subgroup in each model, and a likelihood ratio test was used to compare the nested and full models. We addressed non-linearity in the relationship between BLL and failure rates using a likelihood ratio test for difference in model fit between the adjusted linear BLL model (only linear BLL) with the adjusted non-linear BLL model (linear BLL with quadratic BLL term).

Due to heterogeneity in race (black/white) reporting across Hispanic subgroups (1.3%, 6.3%, and 7.4% black for Mexican-Americans, Puerto Ricans, and Other Hispanics, respectively), race was explored as an effect modifier using the same methods for both linear and log binomial regression models. Race-stratified models were only performed on Puerto Rican children, since there were not enough black Mexican or other Hispanics to do so.

Adjusted population attributable fractions were calculated using log binomial regression models paired with the population attributable and unattributable fractions for cohort and cross-sectional studies (PUNAF) program in Stata. All statistical analyses were performed using Stata/SE 14.0 (StataCorp LP, College Station, TX, USA) [16]. 

## 3. Results

The full-spectrum sample, including those with BLL of 10 µg/dL (0.483 µmol/L) or more, included 13,266 Hispanic children with a mean BLL of 4.93 µg/dL (0.238 µmol/L) and median BLL of 4 µg/dL (0.193 µmol/L). After excluding 947 (7.1%) children with BLL less than 10 µg/dL (0.483 µmol/L), the main analysis sample included 12,319 children with mean BLL of 4.16 µg/ dL (0.201 µmol/L) and median BLL of 4 µg/dL (0.193 µmol/L). Descriptive characteristics and bivariate analysis results, including mean BLLs, mean math and reading scores, and math and reading failure rates, can be seen in Table 1. Mexican children had significantly higher mean BLLs (4.24 µg/dL (0.205 µmol/L)) than Puerto Ricans (4.08 µg/dL (0.197 µmol/L)) or Other Hispanic (3.77 µg/dL (0.182 µmol/L)). BLLs were significantly higher in male children, in children of less educated mothers, and in children from low-income families. Math and reading scores were lowest and failure rates were highest in male children, in Puerto Rican children, in children of less educated mothers, and in children from low-income families. Additionally, math scores were lower and math failure rates were higher in children having very low birthweight or born pre-term. In terms of mean levels, associations were slightly stronger for reading than for math, but for failure rates, associations were slightly stronger for math than for reading.

Compared to linear regression models assuming linear relationships between BLL and testing scores, addition of a quadratic term for BLL did not improve model fit in the reading score model (RMSE_non-linear_ = 13.184; RMSE_linear_ = 13.185) or in the math score model (RMSE_non-linear_ = 12.831; RMSE_linear_ = 12.831). Similarly, addition of quadratic term did not significantly improve model fit in reading failure models (p_LRT_ = 0.25) or math failure models (p_LRT_ = 0.77). Since there was no evidence of a non-linear relationship between BLL and any outcome, blood lead levels were modeled linearly with respect to outcome in all models.

The crude linear regression analysis suggests that in all children, regardless of Hispanic subgroup, both reading and math scores decline significantly with increasing blood lead level (Table 2). Using the fully adjusted model, the test for overall effect of the interaction term between blood lead level and Hispanic subgroup was not significant in either the reading score model (*p* = 0.63) or the math score model (*p* = 0.76), and was thus excluded from the final models. After adjusting for potential confounders, slope estimates were attenuated, but still significantly inversely associated. Specifically, average reading scores declined by 0.55 points and average math scores declined by 0.48 points for each 1 µg/dL increase in blood lead level.

In the crude log binomial analysis, regardless of Hispanic subgroup, both reading and math failure rates were higher in children with higher blood lead levels (Table 3). Using likelihood ratio tests for the fully adjusted model, the model containing the interaction term between blood lead level and Hispanic subgroup did not significantly improve model fit in the reading score model (*p* = 0.96) or the math score model (*p* = 0.91), and was thus excluded from the final models. After adjusting for potential confounders, relative risk estimates were slightly attenuated, but remained statistically significant. For every 5 µg/dL increase in blood lead level, failure rates for reading increased by 43% (relative risks (RR) = 1.43; 95% confidence intervals (CI): 1.25, 1.63), and for math increased by 53% (RR = 1.53; 95% CI: 1.32, 1.78). 

Due to sparse reporting of black race, race (black/white) was not assessed for interaction among Mexican-Americans or other Hispanics. Results for Puerto Ricans, however, suggest a notably stronger association of blood lead level with math failure (*p* = 0.07), but not reading failure (*p* = 0.62), among black Puerto Rican children compared to white Puerto Rican children in adjusted analyses. Specifically, among black Puerto Rican children, we observed a 5.90 (1.45–24.04) times increased risk of math failure for each 5 µg/dL (0.048 µmol/L) increase in blood lead level while among white Puerto Rican children, we observed only a 1.58 (1.13–2.21) times increased risk. The increased risk of reading failure for each 5 µg/dL (0.048 µmol/L) increase was 2.15 (0.62–7.48) and 1.56 (1.14–2.12) for black and white Puerto Rican children, respectively. Results from linear regression models provided similar but non-significant results for both math score (interaction *p* = 0.22) and reading score (*p* = 0.62).

After calculating the adjusted population attributable fractions, we found that for all Hispanic children in our study, 7.0% (95% CI: 1.9–11.9) of all reading failures and 13.7% (95% CI: 7.8–19.2) of all math failures can be attributed to elevated blood lead levels (5–9 µg/dL (0.242–0.435 µmol/L) vs. 0–4 µg/dL (0–0.193 µmol/L)). Since a larger proportion of Mexican-American children have elevated blood lead levels compared to Puerto Rican or other Hispanic children, the proportion of failures attributed to elevated blood lead levels was highest in Mexican-American children (Table 4).

## 4. Discussion

In the present study, we sought to determine the impact of low-level lead toxicity on school performance among Hispanic children, overall and within Hispanic subgroups (Mexican vs. Puerto Rican vs. Other Hispanic). We have demonstrated that among Hispanic children with blood lead levels below 10 µg/dL (0.483 µmol/L), those with higher BLL may be at increased risk for poor performance on standardized tests. We estimated that seven percent of reading failures and almost 14% of math failures among all Hispanic children can be attributed to a BLL of 5–9 µg/dL. While no particular subgroup appears more susceptible to lead, Mexicans do experience slightly higher exposures and have the highest mean blood lead levels, leading them to have a slightly higher proportion of failures attributable to elevated BLL than their counterparts. 

In our earlier study, we found that the impact of low-level lead toxicity on school performance varied by race and ethnicity [13]. Black children had higher mean BLL and poorer performance on ISAT reading and math scores than Hispanics, who in turn had higher mean BLL and poorer performance on ISAT reading and math scores than Whites. After adjusting for covariates, we observed a significant interaction between race/ethnicity and BLL for non-Hispanic Black children compared with non-Hispanic White children, with White children having the steepest declines in ISAT scores per unit increase in BLL. The interaction for Hispanic versus White children was significant for reading, but not math failure. We concluded that these differences were most likely due to unmeasured environmental rather than biological factors.

In the present study, we did not see any significant interactions between BLL and ISAT performance by Hispanic subgroup. We did observe that Black Puerto Ricans showed a steeper decline in scores per unit increase in BLL and failure rates on the math portion of the ISAT test than White Puerto Ricans. This is in contrast to our previous findings, where the declines in performance on the ISAT test per unit increase in BLL were steeper for Whites than for Blacks. The Puerto Rican population are descendants of Spanish, African, and Taino roots, regardless of skin tone. The self-identification of race/ethnicity in this population is complex, and self-classification as Black/White is an oversimplification and prone to misclassification bias [14]. Nevertheless, these findings raise intriguing questions as to the causes for these observed differences in susceptibility.

While BLLs have declined dramatically among U.S. children over the past several decades, environmental health disparities by racial/ethnic groups and socioeconomic status continue to persist. Poor minority children who live in substandard housing continue to be the highest risk group for lead poisoning. The American Housing Survey (2005) showed that 2.9% of Hispanics and non-Hispanic Blacks lived in severely substandard housing compared to 1.6% of Whites [2]. Lead poisoning among Hispanic children is a multidimensional issue that includes environmental, cultural, and social dimensions [4]. In addition to housing, these hazards include exposure from water and soil, parental exposures to lead, pica during pregnancy, lead-glazed pottery used in food preparation, folk remedies such as *greta* and *litargirio*, and imported candy from Mexico [4,6]. 

Hispanic children in the United States also demonstrate ethnic and racial disparities in educational performance [17]. Hispanic children demonstrate consistent underachievement in academic performance from pre-kindergarten through twelfth grade, and are underrepresented in high school graduation rates, placement in gifted and talented programs, and admission rates to postsecondary education, compared to their White American peers. 

Schwartz el al. point out that differences in susceptibility to lead may be due to genetic factors, phenotypic variability, psychosocial stress, and socioeconomic position, and that many of these modifiers covary with exposure [18]. The variability of the effects of lead may also be due to interactions among multiple exposures and transgenerational propagation of risk. The role of genetic factors is unclear, though some studies have suggested susceptibility to the neurotoxic effects of lead may be modified polymorphisms in genes for amino levulinic acid dehydratase, apolipoprotein E, and Vitamin D, or epigenetic mechanisms [18,19,20]. Stress has been shown to exacerbate the neurotoxic effects of lead in animal and human studies [18]. Several studies have also demonstrated that the impact of lead on health is modified by socioeconomic position. The effects of lead are also exacerbated by stress, which may also differ by race and socioeconomic status. Miranda et al. found that the effects of lead on end-of-grade (EOG) testing were more potent in populations at the lower performance regions of the EOG curve, where the effects of other risk factors, such as low socio-economic status and stress, may be more prevalent [4]. Future work is needed, especially among the Puerto Rican population, to further elucidate the effects of these potential effect modifiers. Additionally, future research should continue to explore how environmental factors are packaged in racial subgroups.

The social science literature in the United States consistently demonstrates that race and socioeconomic status are key determinants of educational attainment [21,22,23,24,25,26,27,28,29,30,31]. While these variables clearly interact with each other, race and income determine how well American students do, regardless of the outcome measure (e.g., test scores, graduation rates, work or income levels). Far less work has been done on the environmental factors that are “packaged” in the race and socioeconomic status variables. Exposure to lead is but one of many factors that are embedded, but unmeasured in the variables of race and socioeconomic status. Lead needs to be measured separately so that we have a better understanding of how to disentangle the causality associated with low educational attainment. The last twenty years of educational reform has done little to mitigate the effects of race and socioeconomic status on educational attainment. A large part of the reason for that is our poor measurement of what race and socioeconomic status mean in the lives of children. While the reforms themselves may have had serious weaknesses in terms of both substance and implementation, our understanding of the environmental factors that are poorly measured in the attainment literature add to our ignorance of how race and socioeconomic status “work”. Our study demonstrates a substantial impact of lead on school performance in Hispanic children in the United States after adjusting for poverty, race/ethnicity, gender, maternal education, and very low birth weight or preterm birth.

This study capitalizes on the linkage of existing data across sectors to allow for the study of the effect of BLL on educational outcomes, while controlling for a wide range of relevant confounders. While this was a strength, there are also several limitations of this study. First, although the sample population cohort was large and mostly representative of CPS students, it does not include immigrant children. While the exclusion of immigrant children may reduce generalizability to the US Hispanic population, it is unlikely to introduce selection bias, since immigration precedes test results and thus test results cannot influence immigrant status. Certainly, immigrant status is a likely confounder due to language barriers, but exclusion of immigrants eliminates any bias that would otherwise be introduced. Even among US-born Hispanic children, the language barrier may also act as a confounder of the main associations. Language spoken at home may be associated with elevated BLL as a result of low socioeconomic status and is directly related to poorer test performance. Since we were unable to gather data on language spoken at home, residual confounding may occur as a result of incomplete control of language spoken at home. This would result in exaggerated estimates, but the magnitude of this bias is unknown. Second, different labs reported different limits of detection for BLL, which potentially introduces misclassification of lead exposure. However, misclassification is not likely to differ by test scores or failures, and thus would not introduce misclassification bias. Third, we were not able to adjust for parental IQ or HOME Inventory (a measure of the quality of the home environment), which are typically strong confounders with blood lead exposure. Instead, we relied on surrogate measures, including maternal education and economic status. Finally, the use of free and reduced-price lunch as a proxy for economic status is potentially distorted because there is administrative pressure to maximize program eligibility, which could inaccurately inflate the number of families characterized as living in “poverty.” Use of these surrogate measures over their respective gold standard measures are potential sources of unmeasured or residual confounding and may have resulted in overestimates of effect measures, including PAFs. However, without information on gold standard measurements in this sample, the direction and magnitude of the potential bias is unclear.

## 5. Conclusions

We have demonstrated increased risk of poor performance on standardized tests among Hispanic children with BLL less than 10 µg/dL (0.483 µmol/L). The detrimental effects of lead on school performance were similar for different Hispanic subgroups. Black Puerto Ricans had a higher relative risk of failing the math exam per 5 µg/dL increase in BLL than White Puerto Ricans. Although the current CDC level of concern for lead has been lowered from 10 µg/dL (0.483 µmol/L) to 5 µg/dL (0.242 µmol/L), it is now widely recognized that there is no known safe concentration of blood lead. Many studies have shown that the effects of lead on intellectual function and school performance are non-linear, with steeper drops occurring at lower blood lead levels [13,32]. Hispanics constitute the fastest growing minority population in the United States [33]. They continue to be at risk for environmental health and educational disparities. Given the substantial effects of even low levels of lead on academic performance in all children, the prevention of lead poisoning should continue to be a public health priority.

## Figures and Tables

**Table 1:** Demographic characteristics, mean Blood Lead Levels (BLLs), reading and math ISAT Scores among Hispanic children of the Chicago cohort.

Characteristic	*n* (%)	BLL (µg/dL) Mean ± SD	Reading Score ISAT Mean ± SD	Math Score ISAT Mean ± SD	Reading Failure (%)	Math Failure (%)
Overall	12,319 (100)	4.16 ± 2.03	157.4 ± 13.8	160.5 ± 13.3	9.6	7.5
Gender						
Male	6215 (50)	4.24 ± 2.04 **	156.4 ± 14.1 **	161.1 ± 13.8 **	11.6 **	8.0 *
Female	6102 (50)	4.08 ± 2.01	158.4 ± 13.5	159.9 ± 12.8	7.6	6.9
Hispanic Subgroup ^a^						
Mexican-American	8449 (69)	4.24 ± 2.03	157.6 ± 13.7	160.9 ± 13.3	9.2	6.9
Puerto Rican	2564 (21)	4.08 ± 2.02 **	156.0 ± 14.0 **	158.5 ± 13.2 **	11.9 **	9.7 **
Other Hispanic	1256 (10)	3.77 ± 2.00 **	159.3 ± 14.0 **	162.1 ± 13.5 **	7.6	6.9
Foreign Born Mother						
Yes	7103 (58)	4.17 ± 2.03	157.9 ± 13.7 **	161.6 ± 13.2 **	8.3 **	5.9 **
No	5216 (42)	4.14 ± 2.03	156.7 ± 14.0	159.0 ± 13.4	11.5	9.7
Mother‘s Education ^b^						
Some high school	6609 (54)	4.31 ± 2.06 **	155.9 ± 13.6 **	159.2 ± 13.1 **	11.2 **	8.5 **
High School Graduate	3538 (29)	4.10 ± 2.04	158.0 ± 13.5	161.1 ± 13.1	8.3	6.6
Some College	1671 (14)	3.85 ± 1.90	160.6 ± 13.8	162.8 ± 13.4	7.2	6.0
College Graduate	375 (3)	3.78 ± 1.78	162.3 ± 15.5	165.6 ± 15.1	6.9	6.2
Post College	126 (1)	3.60 ± 1.67	166.7 ± 15.3	169.0 ± 15.4	2.4	2.4
Low-Income						
Yes	10,974 (89)	4.19 ± 2.04 **	156.8 ± 13.6 **	159.9 ± 13.0 **	10.0 **	7.8 **
No	1345 (11)	3.90 ± 1.95	162.4 ± 14.9	165.6 ± 14.6	6.5	4.9
SGA						
Yes	1368 (11)	4.19 ± 2.11	155.5 ± 14.0 **	158.4 ± 13.5	13.1 **	10.8 **
No	10,799 (89)	4.15 ± 2.02	157.7 ± 13.8	160.8 ± 13.3	9.2	7.0
Preterm Birth ^a^						
Term	11,169 (92)	4.17 2.03	157.6 ± 13.8	160.7 ± 13.3	9.3	7.2
Late PTB	765 (6)	4.14 ± 2.07	156.2 ± 14.5 **	159.3 ± 13.1 **	12.8 **	8.4
Early PTB	234 (2)	3.82 ± 1.91 *	154.4 ± 14.3 **	156.2 ± 13.6 **	15.5 **	15.8 **

Note: Cohort includes 12,319 Hispanic Children with BLLs < 10 μg/dL. Summary statistics, including proportions, were calculated among those with non-missing data for that characteristic. Reading and Math test scores are normally distributed as reported by the Illinois Standard Achievement Test (ISAT). Differences in mean blood lead levels and reading and math scores were assessed using standard *t*-test methods; Differences in proportions for reading and math failure were assessed using chi-square tests. ^a^ Nominal variable—linear regression (mean BLL and test scores) and log probability regression (test failure rates) were used to generate multiple *t*-tests with the first category as the reference group; ^b^ Ordinal variable—linear regression (mean BLL and test scores) and log probability regression (test failure rates) were used to generate a single *t*-test for linear trend; * *p* ≤ 0.05; ** *p* ≤ 0.01. SD: standard devation; PTB: preterm birth; SGA: small for gestational age.

**Table 2:** Model summaries for multivariable linear regression models for reading and math ISAT Score (dependent variable) among Hispanic children.

Comparison	All Hispanic Children			Mexican-American Children			Puerto Rican Children		Other Hispanic Children			
	Estimate	SE	*p*	Estimate	SE	*p*	Estimate	SE	*p*	Estimate	SE	*p*
Reading ISAT Score	(*n* = 12,101)			(*n* = 8355)			(*n* = 2507)		(*n* = 1239)			
*Unadjusted* BLL (µg/dL)	−0.72	0.06	<0.001	−0.68	0.07	<0.001	−0.83	0.14	<0.001	−0.74	0.20	<0.001
*Adjusted* ^a,b^ BLL (µg/dL)	−0.55	0.06	<0.001	−0.52	0.07	<0.001	−0.65	0.13	<0.001	−0.62	0.19	0.001
Math ISAT Score	(*n* = 12,081)			(*n* = 8340)			(*n* = 2508)		(*n* = 1233)			
*Unadjusted* BLL (µg/dL)	−0.57	0.06	<0.001	−0.58	0.07	<0.001	−0.51	0.13	<0.001	−0.61	0.19	0.001
*Adjusted* ^a,b^ BLL (µg/dL)	−0.48	0.06	<0.001	−0.50	0.07	<0.001	−0.40	0.13	0.002	−0.54	0.19	0.004

Note: Model Summaries are for Hispanic Children with BLLs Lower than 10 µg/dL, Overall and Stratified by Hispanic Subgroup. The interaction term (BLL*Hispanic subclass) was not included in either model, since the addition of the interaction term into the model did not significantly improve model fit (*p* > 0.05). ^a^ For All Hispanic Children, the model includes blood lead level, gender, mother’s education, low-income, preterm birth, SGA, child’s age at time of BLL, ISAT vs. Iowa, and Hispanic subgroup (Mexican-American vs. other Hispanic and Puerto Rican vs. Other Hispanic); ^b^ For individual races, model includes blood lead level, gender, mother’s education, low-income, preterm birth, SGA, child’s age at time of BLL, ISAT vs. Iowa.

**Table 3:** Log binomial regression models for the effect of Blood Lead Level (BLL) on reading failure and math failure among Hispanic children.

Comparison		All Hispanic Children		Mexican-American Children		Puerto Rican Children		Other Hispanic Children	
		RR	95% CI	RR	95% CI	RR	95% CI	RR	95% CI
Reading Failure		(*n* = 12,101)		(*n* = 8355)		(*n* = 2507)		(*n* = 1239)	
*Unadjusted* BLL (mg/dL)	1 μg/dL increase	1.09	1.06, 1.13	1.09	1.05, 1.13	1.10	1.04, 1.16	1.08	0.98, 1.20
	5 μg/dL increase	1.56	1.35, 1.80	1.55	1.30, 1.86	1.59	1.19, 2.13	1.49	0.90, 2.46
*Adjusted* ^a,b^ BLL (mg/dL)	1 μg/dL increase	1.07	1.05, 1.10	1.08	1.04, 1.12	1.10	1.03, 1.16	1.08	0.97, 1.19
	5 μg/dL increase	1.43	1.25, 1.63	1.47	1.22, 1.77	1.59	1.18, 2.14	1.45	0.86, 2.43
Math Failure		(*n* = 12,081)		(*n* = 8340)		(*n* = 2508)		(*n* = 1233)	
*Unadjusted* BLL (mg/dL)	1 μg/dL increase	1.10	1.07, 1.14	1.10	1.06, 1.15	1.11	1.04, 1.18	1.09	0.98, 1.21
	5 μg/dL increase	1.62	1.38, 1.91	1.63	1.33, 2.00	1.70	1.24, 2.33	1.54	0.91, 2.62
*Adjusted* ^a,b^ BLL (mg/dL)	1 μg/dL increase	1.09	1.06, 1.12	1.09	1.05, 1.13	1.10	1.04, 1.16	1.08	0.98, 1.19
	5 μg/dL increase	1.53	1.32, 1.78	1.53	1.26, 1.85	1.60	1.20, 2.11	1.48	0.90, 2.41

Note: Model Summaries are for Hispanic Children with BLLs lower than 10 µg/dL, overall, and stratified by Hispanic Subgroup. The interaction term (BLL*Hispanic subclass) was not included in either model, since the addition of the interaction term into the model did not significantly improve model fit (*p* > 0.05). ^a^ For All Hispanic Children, model includes blood lead level, gender, mother’s education, low-income, preterm birth, SGA, child’s age at time of BLL, ISAT vs. Iowa, and Hispanic subgroup (Mexican-American vs. other Hispanic and Puerto Rican vs. Other Hispanic); ^b^ For individual races, model includes blood lead level, gender, mother’s education, low-income, preterm birth, SGA, child’s age at time of BLL, ISAT vs. Iowa.

**Table 4:** Population attributable fractions (PAFs) for elevated Blood Lead Levels and Reading/Math ISAT Failure among Hispanic children.

Hispanic Subgroup	Reading Failure		Math Failure	
	PAF (%)	95% CI	PAF (%)	95% CI
All Hispanics ^a^	7.0	1.9, 11.9	13.7	7.8, 19.2
Mexican-American ^b^	7.4	2.0, 12.5	14.5	8.2, 20.3
Puerto Rican ^b^	6.4	1.7, 10.9	12.6	7.1, 17.7
Other Hispanic ^b^	5.8	1.5, 9.8	11.3	6.3, 16.0

^a^ For All Hispanic Children, model used to calculate PAF includes elevated blood lead level (5–9 vs. 1–4 µg/dL), gender, mother’s education, low-income, small for gestational age, preterm birth, child’s age at time of BLL, ISAT vs. Iowa, and Hispanic subgroup (Mexican-American vs. other Hispanic and Puerto Rican vs. Other Hispanic); ^b^ For individual Hispanic subgroups, model used to calculate PAF includes elevated blood lead level (5–9 vs. 1–4 µg/dL), gender, mother’s education, low-income, small for gestational age, preterm birth, child’s age at time of BLL, and ISAT vs. Iowa.
